# Duplications and Losses of the Detoxification Enzyme Glycosyltransferase 1 Are Related to Insect Adaptations to Plant Feeding

**DOI:** 10.3390/ijms25116080

**Published:** 2024-05-31

**Authors:** Jinyu Wu, Wanjiang Tang, Zhengyang Li, Amrita Chakraborty, Cao Zhou, Fei Li, Shulin He

**Affiliations:** 1 College of Life Science, Chongqing Normal University, Chongqing 401331, China; jinyuwu524@163.com (J.W.);; 2 Faculty of Forestry and Wood Sciences, Czech University of Life Sciences Prague, Kamýcká 129, 16500 Prague, Czech Republic; chakraborty@fld.czu.cz

**Keywords:** UDP-glycosyltransferases, insect–plant interaction, feeding niche, herbivory, detoxification

## Abstract

Insects have developed sophisticated detoxification systems to protect them from plant secondary metabolites while feeding on plants to obtain necessary nutrients. As an important enzyme in the system, glycosyltransferase 1 (GT1) conjugates toxic compounds to mitigate their harm to insects. However, the evolutionary link between *GT1s* and insect plant feeding remains elusive. In this study, we explored the evolution of *GT1s* across different insect orders and feeding niches using publicly available insect genomes. *GT1* is widely present in insect species; however, its gene number differs among insect orders. Notably, plant-sap-feeding species have the highest *GT1* gene numbers, whereas blood-feeding species display the lowest. *GT1s* appear to be associated with insect adaptations to different plant substrates in different orders, while the shift to non-plant feeding is related to several losses of *GT1s*. Most large gene numbers are likely the consequence of tandem duplications showing variations in collinearity among insect orders. These results reveal the potential relationships between the evolution of *GT1s* and insect adaptation to plant feeding, facilitating our understanding of the molecular mechanisms underlying insect–plant interactions.

## 1. Introduction

Insects and plants have developed intricate relationships during evolution [1]. Most known insects feed on plants to acquire necessary nutrients [2], while plants employ complex defense mechanisms against insect attacks. Plant defense mechanisms primarily rely on secondary metabolites, which are converted into highly toxically active compounds upon herbivore attack [3]. On the other hand, insects have developed a three-stage detoxification process to protect themselves from toxic compounds. The ingested secondary metabolites are metabolized by detoxification enzymes, such as cytochrome P450, carboxylesterases, and flavin-containing monooxygenases in insects or related microorganisms hosted in insect guts; the toxic by-products are then enzymatically conjugated by glutathione S-transferases and glycosyltransferase; the final products are eventually transported outside the cells and secreted out from insect bodies via binding proteins, such as the ATP binding cassette [4,5,6].

As a key enzyme in the conjugation of toxic chemicals during the detoxification process [7], glycosyltransferase (GT) plays a significant role in both eukaryotic and prokaryotic organisms [8,9]. In insects, GTs are involved in detoxification, tissue development processes, and physiological functions through glycol conjugation [10]. GTs catalyze the formation of glycosidic bonds in polysaccharides and conjugate sugar moieties to acceptors, such as proteins, lipids, and small molecules [11,12]. According to their enzymatic reactions, GTs are classified as inverting or retaining enzymes based on the transferred glycosyl group [13]. In addition, glycosyltransferases can be divided into two major classes, GT-A and GT-B, according to their folding patterns [13]. Based on sequence structure and enzymatic characteristics, GTs are currently grouped into more than 100 families [14,15].

Among them, GT1, widely distributed in bacteria, animals, plants, fungi, and viruses, is the largest family and contains more than 20,000 members [16]. GT1s, commonly referred to as UDP-glycosyltransferases (UGTs), typically utilize UDP sugars as the most common donor [17,18]. All UGTs contain two domains: a highly variable N-terminal substrate-binding domain and a relatively conserved C-terminal sugar-donor-binding domain [19]. The sugar-donor-binding domain, containing around 16 hydrophobic amino acids, including a negatively charged amino acid residue, mainly glutamic acid and aspartic acid, is involved in the binding of UDP moieties of nucleotide sugars [20,21], primarily derived from glucose and containing non-glucose sugar donors such as UDP-Rha, UDP-Gal, UDP-Xyl and UDP-GlcUA [22]. 

GT1s have multiple functional roles in insects. The widely acknowledged function is detoxification via the glycosylation of lipophilic compounds into water-soluble products that are readily excreted or stably managed, thereby protecting the cells from foreign toxic compounds [23]. Detoxification is mainly involved in plant secondary metabolites and insecticides. Several insect GTs have been indicated in insecticide resistance or tolerance, such as *Aphis gossypii* UGTs to sulfoxaflor and bifenthrin susceptibility [6], *A. gossypii* UGT to spirotetramat [24], *Nilaparvata lugens* UGTs to chlorpyrifos and imidacloprid [25,26], and *Plutella xylostella* UGT to chlorantraniliprole [27]. In addition, insect UGTs may play roles in olfaction, endobiotic modulation, and sequestration [9,28]. The notably elevated expression of *UGT* in the antennae of *Drosophila melanogaster*, *P. xylostella*, and *Bombyx mori* has been related to the potential role of GTs in olfaction [29,30,31]. Further evidence has suggested that *B. mori* UGT might be involved in feeding via recognition through their olfactory system [32]. Additionally, UGTs have been involved in the glycosylation or sequestration of multiple physiologically important components, such as cuticle tanning precursors in *Manduca sexta* [33] and dietary flavonoids in *Polyommatus icarus* [19,34]. 

*GT1* genes have been identified in several insects through genome or transcriptome sequencing. The numbers of *GT1* gene copies present in insect species range from 12 (honeybee) to 58 (aphid) [35]. The number of detoxification genes, including *GT1*, is generally higher in omnivorous and herbivorous insects feeding on chemically complex plant tissues than those feeding on relatively simple plant components, such as plant sap, nectar, and pollen [36,37,38]. However, a detailed investigation of the relationship between *GT1* gene copies and insect feeding habits remains to be conducted. Furthermore, as an important detoxification enzyme, how it is related to the shifts in diets in insects remains elusive. Here, we take advantage of 160 publicly available insect genomes to investigate the relationship between feeding types and the duplications and losses of *GT1* genes across various insect groups. 

## 2. Results

### 2.1. The Number of GT1 Genes in Insects

After prediction, we found that some non-insect arthropods, including *Limulus polyphemus*, *Varroa jacobsoni*, and *Centruroides sculpturatus* in the order of Chelicerata, do not have *GT1* genes. Other non-insect species have varied *GT1* gene numbers, ranging from 2 in *Penaeus monodon*, to around 20 in *Dermatophagoides pteronyssinus* and *Daphnia magna*, and to 75 in *Tetranych usurticae* (Supplementary Table S1). 

**Figure 1:**
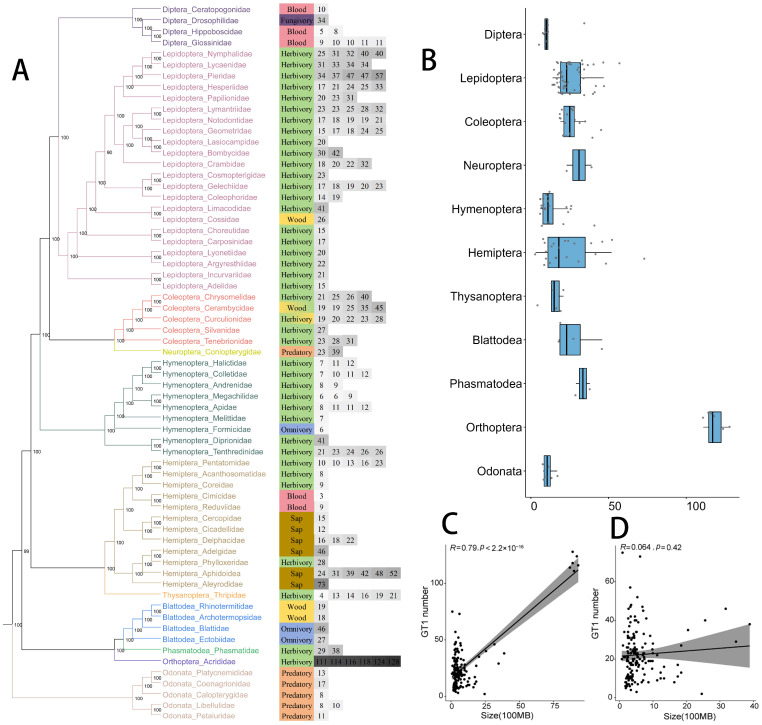
*GT1* gene numbers in 160 insect genomes from different families and orders. (**A**) The left panel is the phylogeny of insect families built by IQ-TREE with single orthologs retrieved from BUSCO, with families and branches in different colors representing different orders. The right panel shows the feeding habits of insect families and predicted *GT1* gene numbers of one or multiple species in the family. (**B**) The *GT1* gene numbers in different insect orders. The correlation between genome sizes and predicted *GT1* gene numbers with (**C**) and without (**D**) species in Orthoptera.

The number of *GT1* genes varies among insect orders, with an average of 25 *GT1* gene copies (Figure 1A). Species in Orthoptera contain the largest *GT1* gene numbers (over 100) among all investigated insects (Supplementary Table S2). Species in Hemiptera have the largest range of *GT1* gene numbers: from 73 in *Bemisia tabaci* to 3 in *Cimex lectularius*, followed by species in Lepidoptera, ranging from 14 to 57 (Figure 1B). Species in the orders Hymenoptera and Coleoptera have relatively stable *GT1* numbers of 6–12 and 19–30, respectively. Species in Diptera and Odonata generally have relatively small *GT1* gene numbers (around 10), except for 34 in *D. melanogaster* in Diptera, the only fungivorous and non-blood-feeding Diptera examined in this study.

Since genome size might influence prediction, we analyzed the correlation between genome assembly size and predicted *GT1* gene numbers. We found a correlation between genome assembly size and the *GT1* gene numbers (Figure 1C). However, this correlation is likely affected by the large *GT1* gene numbers in Orthoptera. Therefore, we excluded them for correlation analysis and found no correlation between the genome assembly size and *GT1* gene numbers across insects except species in Orthoptera (Figure 1D).

**Figure 2:**
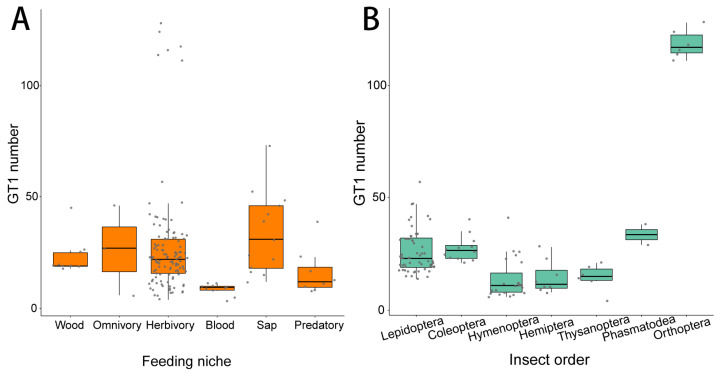
(**A**) The number of predicted *GT1s* at different feeding niches. (**B**) The number of predicted *GT1s* in general herbivorous species of different insect orders.

*GT1* gene numbers also differ with feeding habits. Generally, blood-feeding insects have smaller *GT1* gene numbers, usually fewer than 10 genes (Figure 2A, Supplementary Table S3), compared with sap-feeding species (Kruskal–Wallis test, z = −4.513, *p* < 0.001), general herbivorous species (Kruskal–Wallis test, z = −4.300, *p* <0.001), and wood-feeding species (Kruskal–Wallis test, z = −3.117, *p* = 0.027). Most predatory insects also have few *GT1* genes ranging from 8 to 20, which is obvious but not statistically significantly lower than the *GT1* gene number in sap-feeding insects (Kruskal–Wallis test, z = 2.960, *p* = 0.106). Sap-feeding insects have the largest average *GH1* gene number, followed by omnivores, general herbivores, and wood-feeding insects, although no significant difference has been found between these groups (Figure 2A). 

As general herbivorous insects include species from various orders, we compared the *GT1* gene numbers of general herbivores in different insect orders (Figure 2B). Orthoptera have the largest *GT1* gene numbers among all orders, followed by Thysanoptera. In addition, Coleoptera and Lepidoptera have significantly higher *GT1* gene numbers than Hymenoptera (Lepidoptera vs. Hymenoptera, Z = 4.587, *p* < 0.001; Coleoptera vs. Hymenoptera, Z = 4.079, *p* = 0.001); Coleoptera showed noticeably different but not significantly higher *GT1* gene numbers than Hemiptera (Supplementary Table S4).

In addition, species of different feeding diets in the same insect orders exhibited various *GT1* gene numbers. In Hemiptera, sap-feeding species have more *GT1* gene numbers than blood-feeding (z = −2.649, *p* = 0.024) and general herbivorous species (z = −2.57, *p* = 0.033), although we did not find a significantly difference between the latter two groups (Supplementary Table S5). In Diptera, although we had only one fungivorous species, *D. melanogaster,* its *GT1* gene number is much higher than that of other blood-feeding species.

### 2.2. Phylogenetic Tree of GT1s in Insects and Other Arthropods

**Figure 3:**
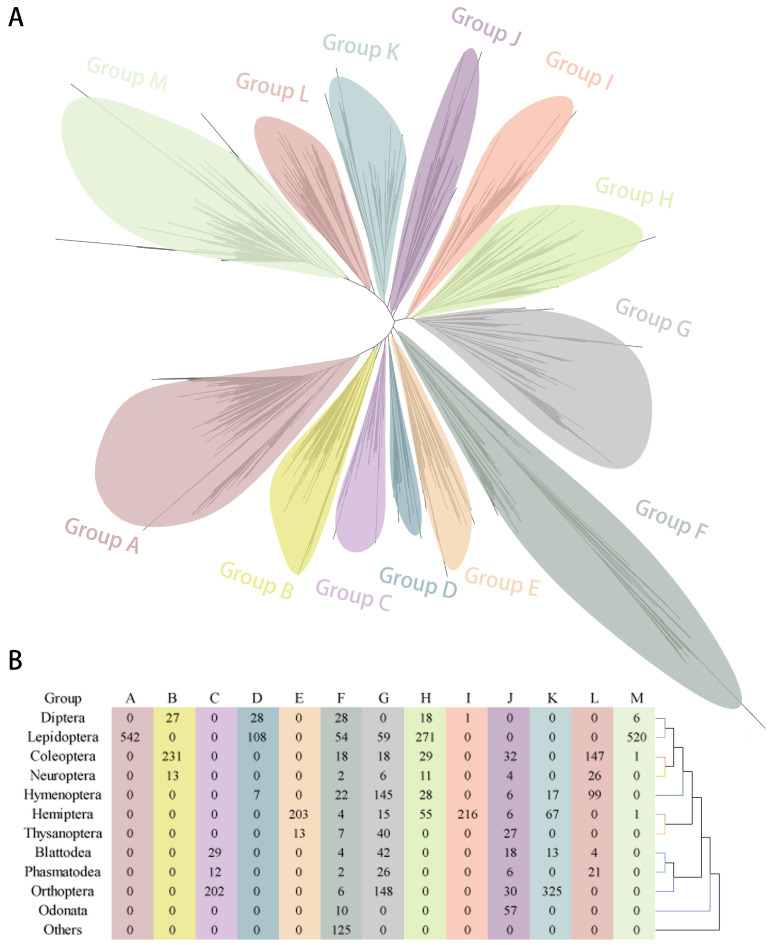
The phylogenetic tree of insect *GT1* genes was constructed using FastTree with the protein sequences of predicted *GT1* genes and visualized using Dendroscope v3.8.10 (**A**). The predicted *GT1s* were categorized into 13 groups based on the phylogenetic tree and summarized in the table by groups and insect orders (**B**).

According to the gene phylogeny (Figure 3A), we clustered insect *GT1s* into 13 groups. Groups A–E and Group I comprised *GT1s* from 1 to 3 insect orders (Figure 3B). Group F was the most diverse group, consisting of *GT1s* from all insect orders and non-insects. Group G and Group J were also diverse groups, but lacked *GT1* from a few insect orders, with Diptera and Odonata in the former and Diptera and Lepidoptera in the latter. Group H and Group M were dominated by *GT1s* of Lepidoptera, but the former also included several *GT1s* of Hemiptera, Hymenoptera, Neuroptera, Coleoptera, and Diptera. Group K and Group L contain *GT1s* from several insect orders. *GT1* genes in the same order tend to cluster together, apart from a few genes that appeared in non-specific order clusters.

### 2.3. Reconciliation between the Gene Tree and Species Tree

**Figure 4:**
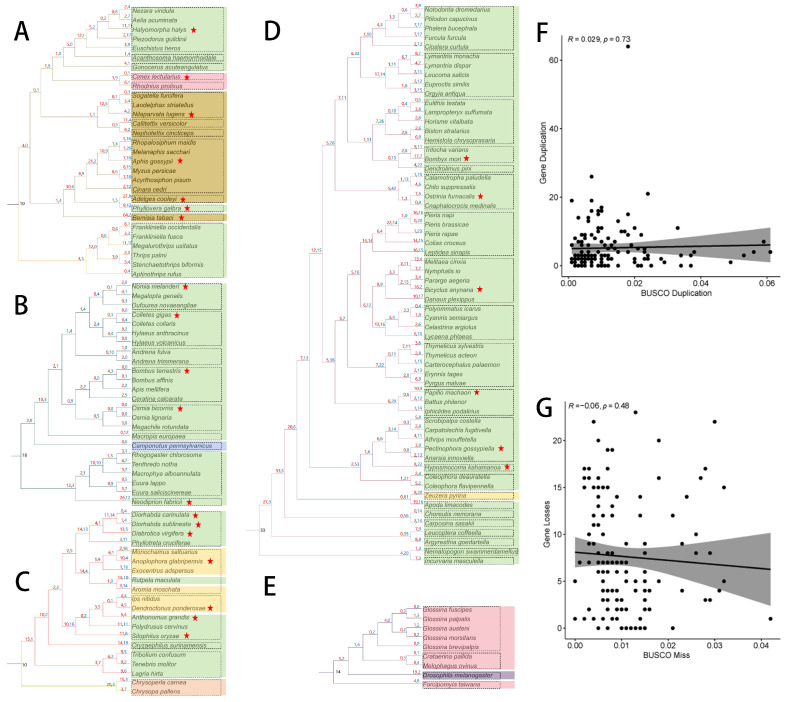
Duplications and losses of *GT1s* inferred by the reconciliation of species trees and *GT1* gene trees of Hemiptera and Thysanoptera (**A**), Hymenoptera (**B**), Coleoptera and Neuroptera (**C**), Lepidoptera (**D**), and Diptera (**E**). The red and blue numbers above the branches indicate duplications and losses, respectively. Species in different colors have different feeding habits: general herbivory in green, wood-feeding in yellow, sap-feeding in brown, blood-feeding in light red, fungivory in light purple, and predatory in light salmon. Different colors of branches indicate different insect orders; dashed boxes indicate insect families in each order. Species labeled with red asterisks were used for subsequent collinear analysis. The correlations between BUSCO duplications and *GT1* duplications (**F**) and between BUSCO missing and *GT1* losses (**G**) at the tips of the species phylogenies.

Due to the large *GT1* gene dataset, we reconciled the *GT1* gene trees with species trees for Hemiptera and Thysanoptera, Hymenoptera, Coleoptera and Neuroptera, Lepidoptera, and Diptera separately. In Hemiptera (Figure 4A), we observed pronounced changes in *GT1* gene number in the one clade containing sap-feeding aphids (up to 21 duplications) and whitefly; *B. tabaci* (64 duplications) compared with the other clade containing blood-feeding bedbug, *C. lectularius*; and sap-feeding brown planthopper, *N. lugens*. In addition, we observed a large number of *GT1* losses (10–20) at the tips of aphids and continual duplications in the internal branches leading to the general herbivorous families.

In Hymenoptera, we generally found a few changes in *GT1* gene numbers with a few duplications and losses, except in the common ancestors and internal branches of sawflies and the common ancestors of bees (Figure 4B).

In Coleoptera and Neuroptera (Figure 4C), we noted generally more duplications than losses, except for a few internal branches. A large number of duplications were observed in the common ancestors of Coleoptera and the branches leading to each family. In addition, the general herbivorous species generally had relatively larger gene number changes than wood-feeding species. Interestingly, we found a large number of duplications in the ancestors of Neuroptera, which are mostly predatory. In Lepidoptera (Figure 4D), we found a large *GT1* gene number (33) in their common ancestors and a large number of duplications in the internal branches close to ancestors; several *GT1* gene losses were identified in most internal branches, especially branches leading to families. In Diptera, we found mostly GT1 gene losses during their evolution except for a large number of duplications (19) in fungivorous Drosophila (Figure 4E). 

To investigate whether the inferred duplications and losses were inflated by the genome assemblies, we analyzed the correlations between BUSCO duplications of genome assemblies and *GT1* gene duplications and between BUSCO missing genome assemblies and *GT1* gene losses at the tips of species phylogenies. We found no correlation in these two comparisons (Figure 4F,G).

### 2.4. Duplication Modes and Collinearity 

To reveal the evolutionary pattern of *GT1* genes in insect genomes, we selected several species with annotations from Hemiptera (7), Hymenoptera (5), Coleoptera (7), and Lepidoptera (7), and inferred the duplication modes for *GT1* genes and analyzed their collinearity in the four orders separately.

**Figure 5:**
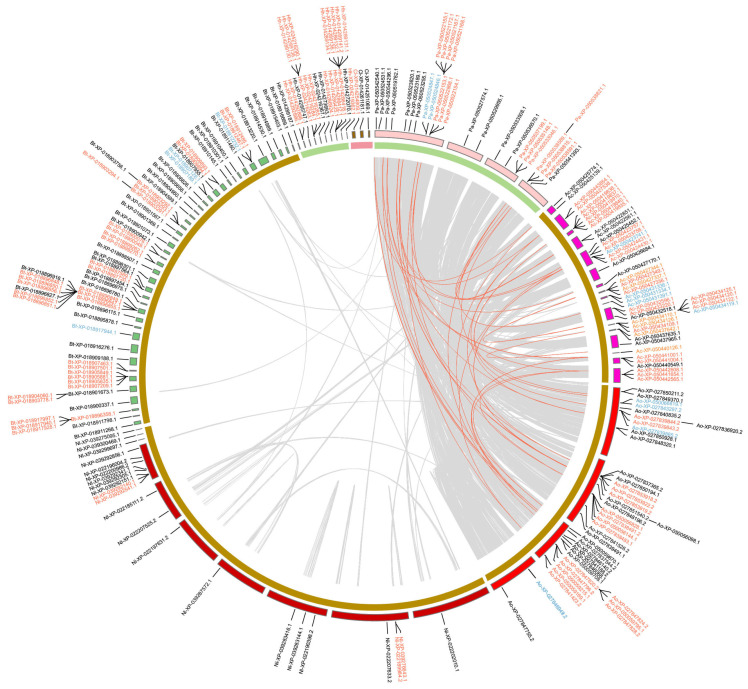
Chromosomal locations and duplication modes of identified *GT1* in selected Hemiptera species. Gene names are presented with two-letter species abbreviations and gene IDs. Ac, *Adelges cooleyi*; Bt, *Bemisia tabaci*; Ao, *Aphis gossypii*; Cl, *Cimex lectularius*; Pa, *Phylloxera galbra*; Nl, *Nilaparvata lugens*; Hh, *Halyomorpha halys*. Red, blue, black, and orange gene IDs represent tandem duplications, proximal duplications, dispersed duplications, and segmental duplications, respectively. Brown, green, and light red curves at the inner circle indicate sap-feeding, general herbivory, and blood-feeding; different colors of karyotypes at the outer circle indicate different species. Connected lines represent collinear blocks between contigs or scaffolds; red lines indicate collinear blocks containing GT1 genes.

**Figure 6:**
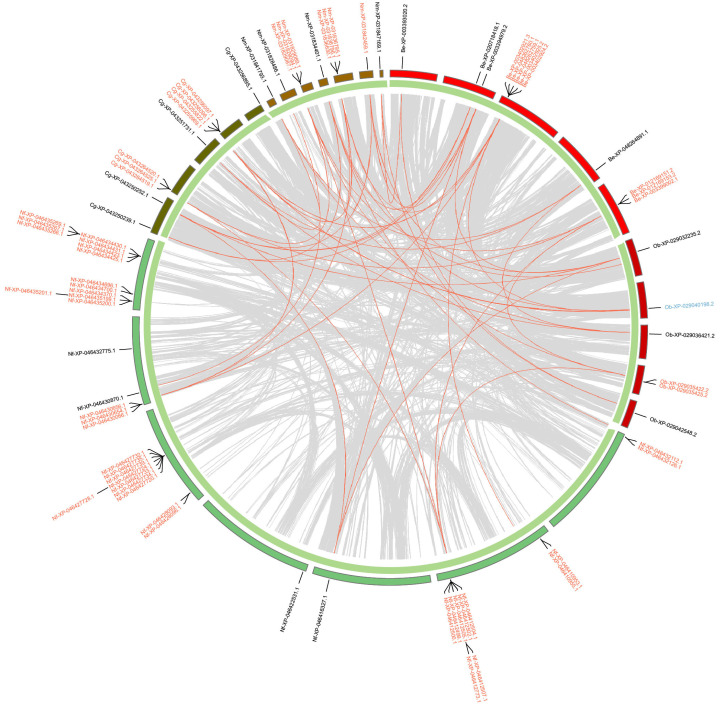
Chromosomal locations and duplication modes of identified *GT1* in selected Hymenoptera species. Gene names are presented with two-letter species abbreviations and gene IDs. Be, *Bombus terrestris*; Cg, *Colletes gigas*; Nm, *Nomia melanderi*; Nf, *Neodiprion fabricii*; Ob, *Osmia bicornis*. Other information is the same as in Figure 5.

**Figure 7:**
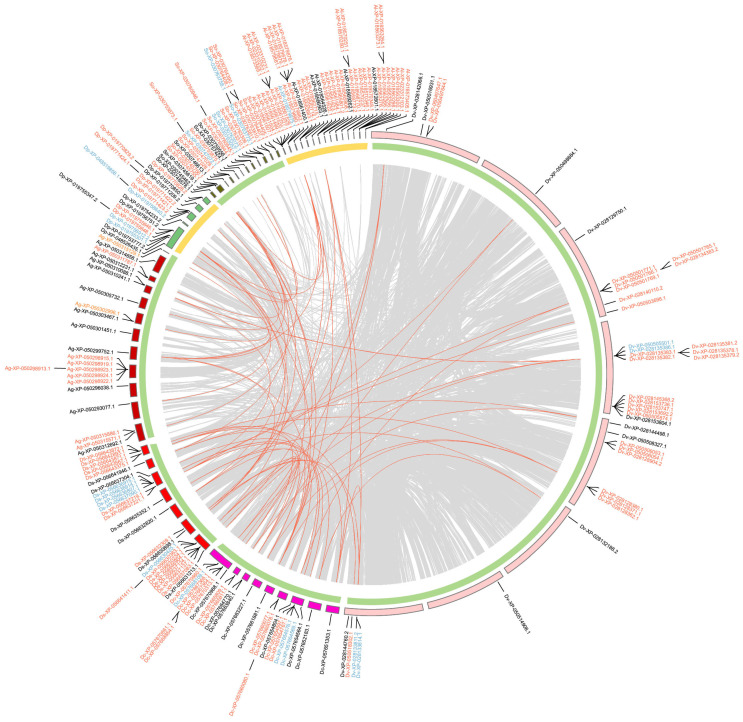
Chromosomal locations and duplication modes of identified *GT1* in selected Coleoptera species. Gene names are presented with two-letter species abbreviations and gene IDs. Al, *Anoplophora glabripennis*; Dv, *Diabrotica virgifera*; So, *Sitophilus oryzae*; Dc, *Diorhabda carinulata*; Ds, *Diorhabda sublineata*; Dp, *Dendroctonus ponderosae*; Ag, *Anthonomus grandis*. Yellow and green curves at the inner circle indicate wood-feeding and general herbivory, respectively. Other information is the same as in Figure 5.

**Figure 8:**
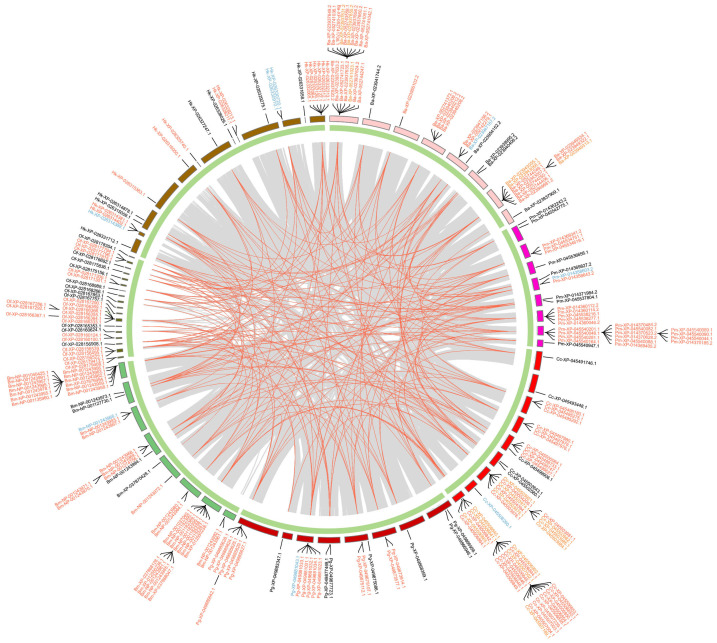
Chromosomal locations and duplication modes of identified *GT1* in selected Lepidoptera species. Gene names are presented with two-letter species abbreviations and gene IDs. Bm, *Bombyx mori*; Hk, *Hyposmocoma kahamanoa*; Of, *Ostrinia furnacalis*; Pg, *Pectinophora gossypiella*; Ba, *Bicyclus anynana*; Pm, *Papilio machaon*; Cc, *Colias croceus*. Other information is the same as in Figure 5.

In Hemiptera (Figure 5), around half of the *GT1* genes are tandem duplications and a few *GT1s* are proximal duplications. Most *GT1s* in the clade of aphids, including *A. gossypii*, *Adelges cooleyi*, and *Phylloxera galbra*, which constitute the majority of tandem *GT1* duplications, are located within collinear blocks. In contrast, most *GT1s* in *B. tabaci* (Bt), *N. lugens* (Nl), *C. lectularius* (Cl), and *Halyomorpha halys* (Hh) are not located within collinear blocks. In Hymenoptera (Figure 6), the majority of *GT1* genes are tandem duplications, most of which, except around half of tandem *GT1* duplications in *Neodiprion fabricii* (Nf), are located in collinear blocks between *Bombus terrestris* (Be), *Colletes gigas* (Cg), *Nomia melanderi* (Nm), and *Osmia bicornis* (Ob). In Coleoptera (Figure 7), over half of the duplications are tandem duplications. Significant *GT1* duplications were identified within collinear blocks in the genomes of *Dendroctonus ponderosae* (Dp), *Diorhabda carinulata* (Dc), and *D. sublineata* (Ds). In addition, many *GT1s* are found in collinear blocks between *Anoplophora glabripennis* (Al), *Sitophilus oryzae* (So), and *Diabrotica virgifera* (Dv); however, around half of these *GT1* tandem duplications are not found in collinear blocks. In Lepidoptera (Figure 8), substantial *GT1s* are tandem duplications located in collinear blocks between these groups. In addition, we found several fragment duplications in *Colias croceus* (Cc, 12); *Bicyclus anynana* (Ba, 6) are found in collinear blocks.

## 3. Discussion

Here, by analyzing the *GT1* genes across 168 publicly available arthropod genomes, we found that *GT1* genes are widely present in insects and have ancient origins at least in the common ancestor of insects.

However, in Chelicerata, *GT1* genes are only present in a few species, such as *Dermatophagoides pteronyssinus* and *T. urticae*, but not in *L. polyphemus*, *V. jacobsoni*, and *C. sculpturatus*. As a herbivore, *T. urticae* has a broad range of plant hosts, which might explain the high number of *GT1* genes, as it is likely to encounter various toxic compounds [39,40]. By determining potential contaminations in the genome assemblies through FCS-GX [41], we found that the contigs or scaffolds containing *GT1s* are from the hosts of *T. urticae* instead of bacteria; however, the *GT1s* are similar to bacterial *GT1s* through BLAST searches, which indicates the possibility that the mite acquired the *UGT* gene from bacteria through horizontal gene transfer, which has been suggested by previous studies [42,43]. Further investigations on the evolution of *GT1s* in non-insect arthropod species are needed to clarify the potential horizontal gene transfers. 

Insects exhibit significant variation in the number of *GT1* genes. Blood-feeding and predatory insects possess much smaller *GT1* gene numbers (11) than plant-feeding insects, including wood-feeding, general herbivory, and sap-feeding (27). A rich repertoire of *GT1s* in the latter group might be related to their adaptation to plant feeding as they encounter a wider range of plant secondary metabolites in the latter group [44,45]; furthermore, this could also be related to the storage and utilization of plant metabolites for defense against predators and parasitoids in some species [46,47]. In addition, some insect herbivores feeding on a large variety of plant species, known as generalists, have a larger number of *GT1* genes, such as *B. tabaci* (73), *Pieris* (average 42), and *Schistocerca* (118); conversely, insects feeding on a narrow diversity of plant species, known as specialists, have fewer *GT1* genes, such as *Aptinothrips rufus* (4), *O. lignaria* (6), and *O. bicornis* (6). This also indicates that the *GT1* gene numbers in plant-feeding insects may be related to the diversity of their feeding substrates as a consequence of the many different sets of chemicals they encounter in various feeding groups [36,48,49]. Notably, we observed significant large *GT1* gene numbers in *Schistocerca* (average 119), which is attributed to their large genomes (average 8.8 G) (Supplementary Table S1) [50].

The duplications and losses of the *GT1* gene family in insects may be related to their adaptation to plant–insect interactions. The significant duplications of *GT1* genes in the ancestors of Lepidoptera, corroborated by the majority of *GT1s* located within collinear blocks, concurred the simultaneous extensive diversification of angiosperms, which is likely related to their adaptation to plant feeding in the ancestors of Lepidoptera [51]. The following extensive tandem duplications and losses throughout the evolution of Lepidoptera suggest close interactions between Lepidopterans and their host plants. In Coleoptera, the duplications of *GT1* genes in their common ancestor might be linked to the emergence of angiosperms as ecological dominants [52]. A large number of duplications in the branches leading to general herbivorous families are likely preadaptations for their adaptation to plant feeding, which is consistent with the diversification of dietary habits in the evolution of beetle families Cerambycidae, Chrysomelidae, and Curculionidae, shifting from mutualistic interactions (pollenivory/pollination) to antagonistic interactions (feeding on various parts of plants) [52]. Interestingly, we also observed large duplications of *GT1s* at the tips of beetle phylogeny, which suggests dynamic interactions between *GT1s* and beetle plant feeding.

In Hemiptera, the variation in *GT1* gene numbers may also be related to the complex evolutionary history of feeding habits. The common ancestors of *B. tabaci* and *Rhopalosiphum maidis* diverged from the ancestors of Hemiptera, which primarily fed on detritus, pollen, fungi, or spores [53] with a small number of *GT1s*, at an early stage [54], and then shifted to vascular plant feeding after diverging into sawflies and aphids, concurring large *GT1* duplications. Subsequently, species in aphids underwent a large number of *GT1* losses as they adapted to different host plants [53], which is supported by a relatively stable GT1 repertoire located in collinear blocks between these aphid species. In another branch of Hemiptera, the common ancestor of Heteroptera underwent a dietary shift from general herbivory to predation, concurring with the loss of *GT1* genes in their common ancestors [53] and resulting in a few *GT1* gene copies in blood-feeding species, *C. lectularius* and *Rhodnius prolixus*. The remaining Heteroptera reverted to general herbivory, accompanied by a small number of *GT1* gene duplications. These shifts in feeding habits, accompanied by the duplications and losses of *GT1* genes, suggest a correlation between *GT1* gene evolution and plant feeding in Hemiptera. 

The common ancestor of Hymenoptera primarily fed on plants. Tenthredinoidea diverged from other Hymenopterans at an early stage and adapted to a wide range of hosts, and continuously gained *GT1* genes during evolution [54]. The other members of Hymenoptera first transitioned from general herbivory to carnivory, which concurred with several *GT1* gene losses [55]. Anthophila originated coincidentally with the diversification of angiosperms, and their ancestors gradually shifted from carnivorous diets to herbivores that fed on pollen and nectar [55,56].

In addition to the copy number, their expression levels of *GT1* might contribute to the adaptation of insects to plant feeding. Although we did not investigate the expression of *GT1* genes, previous studies have suggested that the varied expression of *GT1* genes across different insect tissues probably has different functions. Most GT1 genes are highly expressed in the Malpighian tubules and midgut and are often associated with detoxification [4,57]. However, some *GT1s* are highly expressed in other tissues and might be related to different functions, such as in antennae for odor degradation [29,58] and in the head to synthesize endogenous substances [4]. However, further studies on *GT1* gene expression are needed to elucidate the relationship between *GT1* functions and insects.

Overall, by identifying the *GT1* genes in 168 arthropod species and initially investigating the relationship between insect diets and the abundance of *GT1* genes, we reveal the correlation between duplications and losses of *GT1* and insect adaptation to plant feeding. These results would not only help us understand the evolution of insect detoxification enzyme *GT1* in insect adaptation to plant feeding but also shed new light on the evolution of insect–plant interactions.

## 4. Materials and Methods

### 4.1. Data Collection

In this study, we retrieved 248 publicly available insect genomes from the NCBI GenBank database (accessed on 22 August 2023). Subsequently, we evaluated the quality of the insect genome assemblies by using BUSCO v5.4.3 with the “insecta_odb10” dataset and filtered out low-quality assemblies with fewer than 95% complete BUSCO scores from the subsequent analyses [59]. As a result, 160 species, including 4 in Blattodea, 23 in Hemiptera, 60 in Lepidoptera, 24 in Hymenoptera, 18 in Coleoptera, and 9 in Diptera, were selected for further analyses. Additionally, we downloaded 8 genome assemblies from non-insect arthropods (Supplementary Table S1). 

The feeding niches of the selected insects were obtained from a previously published study [60]. These niches were determined by their feeding materials as in the published study; the feeding behaviors were grouped into 7 categories: sap-feeding, blood-feeding, wood-feeding, omnivory, general herbivory, fungivory, and predatory. General herbivores include insects feeding on various non-vascular plant tissues, such as leaves, flowers, fruits, seeds, and/or root tips. For species whose feeding habits are uncertain, we determined their feeding habits by manual search [37,61,62,63,64,65,66]. 

### 4.2. GT1 Gene Identification

We identified *GT1s* for reference genomes and non-reference genomes separately. For reference genomes retrieved from the Refseq database, we retrieved the longest isoforms for each gene based on the annotation gff file and proteomes using the orthologr package v0.4.0 [67]. The longest isoforms were subjected to *GT1* identification using run_dbcan v4.0.0, which performs three different prediction tools: HMMER, Diamond, and dbCAN_sub [68]. The predictions were considered confident when the three tools produced the same prediction. Subsequently, the predicted protein sequences were combined and clustered by CD-HIT v4.8.1 to construct a non-redundant GT1 database for subsequent predictions on non-reference assemblies [69,70,71].

For the non-reference assemblies, we first predicted *GT1* genes by homologous prediction using genblastG v1.0.138, which builds high-quality gene models by combining the HSPs of blast queries against databases [72]. The predicted gene models were then further curated by filtering incomplete CDS sequences without start and stop codons and removing redundancy with GffRead v0.12.7 [73]; for overlapping *GT1* gene model candidates at the genome location, the gene model with the highest genblastG score was selected. Subsequently, the CDS and protein sequences of predicted *GT1* gene models were retrieved for further *GT1* prediction by run_dbcan [68], following the same annotation procedure for reference genomes. 

To remove any potential contamination of *GT1s* from a non-host origin, such as bacteria, during genome sequencing, we determined the contamination of genome scaffolds or contigs containing predicted *GT1s* by the NCBI foreign contamination screen (FCS) v0.5.0. 

### 4.3. Gene Tree Inference

To infer a gene tree of *GT1s* in insects, we aligned the predicted GT1 protein sequences by using MAFFT v7.520 [74], MUSCLE v5.1 [75], and Clustal Omega v1.2.4 [76]. The alignments were further refined with RASCAL v1.34 [77]. The refined and original alignments were scored using normd v1.2 and the alignment with the highest score was used to construct a gene tree using FastTree v2.1.11 [78]. 

### 4.4. Gene Duplication and Loss Inference

To reveal the changes in *GT1* gene numbers during insect evolution, we inferred gene duplications and losses by reconciling gene trees with a species tree using notung v2.9.1.5 [79]. We inferred the duplications and losses for five major orders individually, including Hemiptera and Thysanoptera, Hymenoptera, Coleoptera and Neuroptera, Lepidoptera, and Diptera. To obtain highly confident gene trees, we inferred a *GT1* gene tree for each order separately with a similar alignment procedure and iqtree v2.2.0. The insect species tree was constructed using a universal single-copy orthologs (USCOs) gene set retrieved from BUSCO; the protein sequences of USCOs were aligned by MAFFT, trimmed by trimAl v1.4, and concatenated by FAsconCAT v1.05.1 into a matrix; the matrix was then subjected to species tree construction by maximum likelihood inference, implemented in iqtree2 [80]. 

### 4.5. Duplicate Mode Inference and Collinearity Analysis

We selected species that had annotation files with mostly chromosome-level assemblies in Lepidoptera, Coleoptera, Hemiptera, and Hymenoptera for collinear analysis. The duplication mode of *GT1* genes was inferred for each species using duplicate_gene_classifier in MCScanx. The collinear analysis was conducted with MCScanx [81] for each order separately, and the output was plotted using circos v0.69-9 [82].

### 4.6. Statistical Analysis

To compare the *GT1* gene numbers in different feeding groups, we conducted pairwise comparisons using the Kruskal–Wallis test with Dunn post hoc tests (R package FSA v0.9.5) [83]. The *p*-values of multiple comparisons between groups were adjusted using the Bonferroni correction. The phylogeny of insect families used in this analysis was inferred from previous analysis (Figure 1A).

## Data Availability

All data associated with the study are publicly available.

## Supplementary Materials

The following supporting information can be downloaded at: https://www.mdpi.com/article/10.3390/ijms25116080/s1.
